# Research progress of different components of PM_2.5_ and ischemic stroke

**DOI:** 10.1038/s41598-023-43119-5

**Published:** 2023-09-25

**Authors:** Bin Li, Yong Ma, Yu Zhou, Erqing Chai

**Affiliations:** 1 First Clinical Medicine College, Gansu University of Traditional Chinese Medicine, Lanzhou, 730000 China; 2 Ningxia Medical University, Yinchuan, 750000 China; 3 Lanzhou University, Lanzhou, 730000 China; 4 Key Laboratory of Cerebrovascular Diseases of Gansu Province, Cerebrovascular Disease Center, Gansu Provincial People’s Hospital, Lanzhou, 730000 China

**Keywords:** Neurological disorders, Atmospheric chemistry, Environmental impact, Diseases of the nervous system

## Abstract

PM_2.5_ is a nonhomogeneous mixture of complex components produced from multiple sources, and different components of this mixture have different chemical and biological toxicities, which results in the fact that the toxicity and hazards of PM_2.5_ may vary even for the same mass of PM_2.5_. Previous studies on PM_2.5_ and ischemic stroke have reached different or even opposing conclusions, and considering the heterogeneity of PM_2.5_ has led researchers to focus on the health effects of specific PM_2.5_ components. However, due to the complexity of PM_2.5_ constituents, assessing the association between exposure to specific PM_2.5_ constituents and ischemic stroke presents significant challenges. Therefore, this paper reviews and analyzes studies related to PM_2.5_ and its different components and ischemic stroke, aiming to understand the composition of PM_2.5_ and identify its harmful components, elucidate their relationship with ischemic stroke, and thus provide some insights and considerations for studying the biological mechanisms by which they affect ischemic stroke and for the prevention and treatment of ischemic stroke associated with different components of PM_2.5_.

## Introduction

Particulate matter 2.5 (PM_2.5_) refers to particulate matter in the atmosphere with an aerodynamic diameter less than or equal to 2.5 μm, which currently ranks 6th in terms of its contribution to the global burden of disease and has become an environmental risk factor that poses a serious threat to public health^1^. Although PM_2.5_ particles are small in size, PM_2.5_ is widely considered to be more toxic. On the one hand, PM2.5 is more likely to be deposited in the respiratory tract than other pollutants of the same mass and can even be directly transferred to the brain because PM2.5 particles are smaller and can be suspended in the air for a longer period; on the other hand, PM2.5 can enrich the air with harmful substances and adsorb more toxic chemicals because of its larger surface area/mass ratio^2^. The composition of PM_2.5_ is complex, and the physical structure, chemical composition, and source of its components are different, resulting in its pathogenic mechanism is not clear, which determines that it is difficult to best quantify the most harmful aspects of PM_2.5_ to health from the level of PM_2.5_, which requires us to conduct health risk assessment at the level of PM_2.5_ components. As a compound pollutant, PM_2.5_ contains various toxic components^3^, including water-soluble inorganic ions, carbon-containing components, inorganic elements and organic matter, etc.^4^. In addition to the above-mentioned chemical-related components, some studies have also shown that PM_2.5_ also contains microorganisms^5^.

Stroke is known to be one of the leading causes of death and disability for patients worldwide and is a serious threat to human health as a neurological disease^6^. There are two main subtypes of stroke, namely ischemic stroke (IS) and hemorrhagic stroke. IS is caused by the loss of blood supply to a region of the brain due to occlusion of an intracranial artery or embolism of an embolus originating from a heart or neck vessel caused by progressive atherosclerosis, and is a common type of stroke, accounting for 87% of the total incidence of stroke^7^.

In studies on the global burden of disease, it is estimated that approximately 4.7 million people died from PM_2.5_ in 2015^8^ and that 30% of stroke risk globally can be attributed to air pollution^9^, thus leading to an increasing interest in PM_2.5_-related IS. Some studies suggest that short-term exposure to PM_2.5_ may increase morbidity and mortality from IS^10^; others suggest that long-term exposure to ambient PM_2.5_ is associated with premature death from IS^11^. In summary, although epidemiological studies around the world have shown that exposure to PM_2.5_ leads to a sustained increase in the morbidity and mortality of IS, related studies have also pointed out that the association estimates between PM_2.5_ and IS show spatial and temporal heterogeneity^12^. There is growing evidence that differences in PM_2.5_ chemical composition contribute to differences in PM_2.5_ toxicity^13^, as different components of PM_2.5_ may have different effects on IS, and previous studies have mostly focused on the total mass of PM_2.5_, so this may have contributed to the heterogeneity of the findings, however, the evidence is limited and further studies are needed^14^. It has also been explored which chemical components of PM_2.5_ have a greater impact on IS, but only a limited number of components have been examined and conclusions remain inconsistent^15^. Therefore, this paper summarizes and organizes a large amount of literature on PM_2.5_-related IS-related literature and sorts out the associations between different PM_2.5_ components and IS, which will help researchers understand the characteristics of toxic particles in PM_2.5_, identify the most harmful PM_2.5_ components, and gain insight into the relationship between these components and IS, thus guiding for identifying susceptible individuals and reducing their exposure to PM_2.5_ exposure, and also provide evidence for the development of air pollution management policies.

To understand the correlation and research progress between PM_2.5_ and its different components and ischemic stroke disease, we searched for relevant literature in the Pubmed database, with a search period from the database establishment to March 31, 2023. We use keywords for relevant searches, mainly including "ischemic stroke" and "PM_2.5_"; Secondary keywords include "water-soluble inorganic ions", "carbon-containing fractions", "inorganic elements", "organic matter", and "microorganisms". The retrieved literature was selected and included in our study based on whether PM_2.5_ and its related components were studied in the ischemic stroke population by reading the title and abstract.

## The relationship between different components of PM_2.5_ and IS

### Water-soluble inorganic ions

Water-soluble inorganic ions are one of the most important components of PM_2.5_, accounting for about 30% to 50% of PM_2.5_ mass concentration^16^. Water-soluble inorganic ions, also known as water-soluble anions and cations, are divided into cations such as ammonium (NH_4_^+^), calcium (Ca^2+^), potassium (K^+^), sodium (Na^+^), magnesium (Mg^2+^) and anions such as sulfate (SO_4_^2−^), chloride (Cl^−^), nitrate (NO_3_^−^), fluoride (F^−^), and bromide (Br^−^) due to their different charged properties^17^. Among all water-soluble inorganic ions in PM_2.5_, three secondary ions (sulfate-nitrate-ammonium salts, SNA) are the most abundant^18^. Sulfate is a salt formed by SO_4_^2−^ from the precursor sulfur dioxide (SO_2_) and related ions, and there is a correlation between sulfate and IS. It has been found that sulfate can enter the body through the airway and blood circulation, causing platelet aggregation and vascular endothelial cell damage, thus increasing the risk of IS in patients; the main sources of urban nitrogen oxides (NO_X_) are internal combustion engines and the combustion of coal, oil, natural gas, and wood, which can undergo complex chemical reactions with various substances in the atmosphere to form NO_3_^−^, and the generated NO_3_^−^ can form nitrates with related ions, and there is a certain correlation between nitrates and IS; ammonium salts are salts formed by NH_4_^+^ and related ions, which are also an important component of PM_2.5_, and there is a certain association between ammonium salts and IS, which can increase the risk of developing IS by stimulating the proliferation of blood vessel wall cells through chronic inflammation and oxidative stress, leading to blood vessel narrowing^19^.

Zhou et al. ^20^conducted a retrospective study of 100 patients with chronic obstructive pulmonary disease (COPD) from August 2014 to September 2019 in Shanghai, China, and other locations, and obtained daily PM_2.5_ and PM_2.5_ fractions from sentinel monitoring stations. The study aimed to investigate the relationship between short-term exposure to different chemical components of PM_2.5_ and cardiopulmonary function indices in COPD patients, and it was found that water-soluble ions in PM_2.5_, mainly NO_3_^-^, SO_4_^2-^ and NH_4_^+^, were closely associated with decreased left ventricular ejection fraction, and this study suggests that water-soluble ions in PM_2.5_ may be an important component associated with decreased cardiopulmonary function in COPD patients. Although this study was conducted in COPD patients, this study suggests that we should pay attention to the hazards of the water-soluble inorganic ion component associated with PM_2.5_ and conduct relevant studies to explain its pathogenic mechanisms. Zhang et al. ^21^ obtained city-specific daily numbers of hospitalizations for cardiovascular disease (CVD) in 18 cities in China between 2014 and 2017 through the Chinese National Basic Medical Insurance Database for Urban Employees and the Beijing Municipal Health and Family Planning Commission Information Center database and collected directly measured PM_2.5_ components through the Chinese Environmental Public Health Tracking System, including ions and polycyclic aromatic hydrocarbons (PAHs). This study aimed to investigate the association between short-term exposure to PM_2.5_ components and hospitalization for CVD. The study found generally consistent correlations between ions and IS, for example, NH_4_^+^ was associated with IS, and the study showed that for subtypes of CVD, the risk of hospitalization varied by exposure to different PM_2.5_ components, with those exposed to NH_4_^+^ having the highest risk of ischemic stroke. Rodins et al.^22^ used baseline (2000–2003) and 14-year follow-up data from the Heinz-Nixdorf Recall study based on a prospective cohort study in a West German population to assess the effect of long-term exposure to specific pollutants and components of Particulate matter (PM) on the incidence of stroke, coronary heart disease, and total cardiovascular events The impact of long-term exposure to specific pollutants and PM components on the incidence of stroke, coronary heart disease, and total cardiovascular events. This study showed that long-term exposure to environmental source-specific air pollution and its chemical components was associated with increased stroke risk and that the PM components SO_4_^2-^ and NH_4_^+^ showed stronger effects than the other components. Liu et al. ^23^based their study on a longitudinal survey of 14,331 adults across 25 provinces and regions in China that began in 2010 to quantify the relationship between CVD incidence and long-term exposure to PM_2.5_ and its components. The study found that the risk ratio for total CVD (including stroke) was associated with increased exposure to PM_2.5_ mass; increased CVD risk was also significantly associated with several PM_2.5_ fractions, the largest being SO_4_^2−^, followed by NH_4_^+^, NO_3_^−^, and black carbon (BC). In summary, long-term exposure to PM_2.5_ and specific components (i.e., BC, NO_3_^−^, NH_4_^+^, and SO_4_^2−^) was associated with an increased risk of total CVD development in Chinese adults, a finding that may be important for a deeper understanding of the biological mechanisms underlying the chronic effects of environmental PM_2.5_ on cardiovascular health. Zanobetti et al.^24^ evaluated the association between daily PM_2.5_ and hospital admissions for diseases such as CVD in 26 communities in the United States between 2000 and 2003, intending to explore whether PM_2.5_ chemistry changed the association between cause-specific hospital admissions and PM_2.5_. The study found that for every 10 μg/m^3^ increase in PM_2.5_ concentration, CVD increased by 1.89%; the association between PM_2.5_ and CVD admissions was significantly altered at high-quality Na^+^. The study showed that higher Na^+^ mass in PM_2.5_ significantly increased its effect on CVD admissions.

### Carbon-containing components

The carbon component of PM_2.5_ is mainly composed of organic carbon (OC), elemental carbon (EC), and carbonate carbon (CC), which account for about 20–50% of the mass concentration of PM_2.5_, with OC and EC accounting for about 10–70% of the mass concentration of the carbon component of PM_2.5_, and CC accounting for a small percentage^25^. OC includes aliphatic, aromatic, PAHs, alkanes, organic acids, etc., and is further divided into primary organic carbon (POC), which is directly emitted by pollution sources, and secondary organic carbon (SOC), which is generated by complex reactions. OC is mainly generated by vehicle exhaust and other emissions, and research shows that OC can enter the body through the airway and blood circulation, and regulate a variety of hormones and cell receptors, accelerate plaque formation and platelet increasing the risk of IS; EC is a single-matter carbon directly emitted by incomplete combustion of biological or fossil fuels with stable chemical properties, EC is also known as light-absorbing carbon or BC^26^, which is the main absorber of visible solar radiation in the atmosphere, and vehicle emissions are its main source^27^; CC is not discussed in the analysis because of its low content in particulate matter and its stable nature^28^.

Previous studies have shown that the implementation of air quality policies and economic changes in New York State from 2008 to 2013 led to decreases in PM_2.5_ and its major constituents (EC and OC) concentrations, decreases in POC concentrations, and increases in SOC concentrations^29^; more recently, a study by Zhang et al. ^30^found that from 2005 to 2016, a small but significant increase in IS hospitalization rates in urban centers in New York State was associated with short-term upward changes in ambient PM_2.5_ concentrations. Taken together, these findings suggest that changes in PM_2.5_ composition caused by policy and economic factors may have triggered IS to varying degrees. Similarly, the Rich et al. ^31^study on specific fine particle concentrations triggering cardiovascular hospitalization in urban centers in New York State analyzed the relationship between acute cardiovascular event hospitalization rates and the average contribution of individual PM_2.5_ sources over the previous 1, 4, and 7 days, and found that increased spark ignition emissions (GAS) concentrations were associated with increased IS hospitalization rates and that when GAS-related PM_2.5_ toxicity increased, only SOC concentrations were increasing, so additional exposure to SOC and associated oxidative stress and inflammation would be a potential driver of increased hospitalization rates. Aturinde et al. ^32^ studied the association between multiple pollutants (this included BC) and CVD incidence across Sweden and found a weak but significant association between the BC component of air pollutants and CVD admissions and suggested that this could be a potential risk factor for IS, atherosclerotic disease. Vivanco-Hidalgo et al. ^33^ explored the relationship between short-term exposure to outdoor ambient air pollutants BC and IS and its different subtypes based on Barcelona. The study did not find a statistically significant association between pollutant exposure and overall ischemic stroke risk, but in a subtype analysis, the duration of exposure 24–47 h and 48–72 h before stroke was found to be associated with large atherosclerosis subtypes of stroke. This study suggests that although no association was found between BC exposure and AIS risk, large atherosclerotic stroke may be triggered by a daily increase in BC of diesel-related pollutants in the study area, which provides ideas and implications for studies of short-term exposure to BC causing ischemic stroke in different subtypes. Ljungman et al. ^34^studied the relationship between long-term exposure to air pollution and IS in three Swedish cities (Gothenburg, Stockholm, and Umesa), and overall, long-term exposure to BC in an environment with low pollution levels was associated with an increased risk of IS, but there was little consistent correlation between the remaining components of PM_2.5_ and IS. The reason for this result may be that the relatively low exposure level is one of the reasons for the lack of association. In a Swedish-based cohort study, Carlsen et al. ^35^ explored the relationship between long-term exposure to source-specific air pollutant BC for the occurrence of CVD, but in contrast to the previously described study, the Carlsen et al. study found no association between exposure to BC and IS. This different finding may be related to the location chosen for the study, where the population was at low exposure levels of air pollution. BC may be an important air pollution indicator to further guide air quality control policies and may have a broader impact than other ambient traffic particulate matter. Liang et al. ^36^ conducted a prospective cohort study of 90,672 adults aged 18 years or older in 161 districts and counties in China from 2010 to 2017, aiming to investigate the association between long-term exposure to PM_2.5_ and its components and CVD mortality in China and to assess the long-term cardiovascular effects of PM_2.5_ and its components, finding that in the general population in China, long-term exposure to PM_2.5_ and its components were associated with an increased risk of cardiovascular mortality, with hazard ratios (95% confidence intervals) of 1.02 (1.00, 1.05) for overall CVD, 1.03 (1.00, 1.06) for overall stroke, and 1.11 (1.04, 1.19) for IS for each 10 μg/m^3^ increase in PM_2.5_ concentration; the PM_2.5_ component hazard study showed that PM_2.5_ components from fossil fuel combustion (i.e., BC, etc.) show a greater hazard ratio than total PM_2.5_ mass. These findings may provide new scientific evidence for the development of regulatory policies and standards and for the assessment of disease burden, particularly in developing countries. Avellaneda-Gómez et al.^37^ used data from the Administrative Health Registry of the Catalan Public Health System in Spain to construct a cohort of people aged 18 years and older without a stroke diagnosis before January 1, 2016, and then collected data on sociodemographic characteristics and cerebrovascular risk factors, and derived PM_2.5_, BC, and other indicators for participants whose residences were within a 300-m buffer zone, BC, and other indicators, The study found that individuals with higher exposure to air pollution in their residential area had a higher risk of IS. Jhun et al. ^38^ synthesized studies conducted in the Boston area on the cardiovascular health effects of traffic exposure, specifically defined as BC or particulate matter number (Pn) exposure or proximity to major roadways, and showed that exposure to traffic-related particulate matter adversely affects cardiac autonomic function, increases systemic cytokine-mediated inflammatory and thrombogenic activity and increases hypertension and IS risk, there is compelling evidence across multiple exposure metrics (e.g., short- and long-term, observational and simulated) and different population cohorts (e.g., elderly, individuals with co-morbidities, young healthy individuals) that BC and Pn represent traffic-related particulate matter that is particularly detrimental to cardiovascular health, Most notably, compared with PM_2.5_ exposure, the cardiovascular risks of Pn and BC exposure were estimated to be greater in magnitude or more statistically significant. Peng et al. ^39^ used a national database that included data on emergency hospital days for cardiovascular and respiratory outcomes from 2000 to 2006 and also collected data on ambient levels of major PM_2.5_ chemical constituents, such as sulfate, nitrate, silicon (Si), EC, OC, Na^+^, and NH_4_^+^, as well as weather information, with the aim of investigating the relationship between hospitalizations for CVD and respiratory disease in the United States relationship with PM_2.5_ chemical composition. The study showed that ambient levels of EC and OC, primarily from vehicle exhaust, diesel, and wood combustion, were associated with the greatest risk of emergency hospitalization among the major chemical constituents of PM_2.5_. Krall et al. ^40^analyzed the seven components of PM_2.5_: OC, EC, Si, Na^+^, and SNA, using data from the US EPA's Chemomorphic Network, and evaluated the short-term relationship between non-accidental mortality and PM_2.5_ components in conjunction with non-accidental mortality in 72 urban communities in the US between 2000 and 2005, in a study designed to provide the first national, seasonal-specific, and region-specific associations between mortality and PM_2.5_ components. Increases in OC, EC, Si and Na^+^ were found to be associated with increased mortality; the study suggests that some components of PM_2.5_ may be more toxic than others.

### Inorganic elements

The inorganic elements in PM_2.5_ are mainly aluminum (Al), magnesium (Mg), calcium (Ca), titanium (Ti), mercury (Hg), copper (Cu), zinc (Zn), lead (Pb), silver (Ag), cadmium (Cd), beryllium (Be), arsenic (As), cobalt (Co), iron (Fe), manganese (Mn), molybdenum (Mo), selenium (Se), thallium (Tl), bromine (Br), chromium (Cr), vanadium (V), and nickel (Ni). Cr), vanadium (V), and nickel (Ni), the types and contents of which vary according to climate, season, geographical location, different pollution sources, and combustion products^41^. Heavy metals are the main inorganic elements in PM_2.5_. Existing studies show that most heavy metals, especially those from anthropogenic pollution sources, are more likely to be enriched in PM_2.5_, which are generally not easily degradable and bioconcentrated, and can cause irreversible damage to several human organs and tissues^42^. Heavy metal elements contained in PM_2.5_ account for about 1.28% to 5.74% of PM_2.5_ mass, and are classified according to their content as major elements (e.g., Al, Mg, Ca, etc.), minor elements (e.g., Ti, Cu, Zn, etc.) and trace elements (Be, Ag, Cd, etc.); they are classified according to their sources as crustal elements and pollution elements, with crustal elements (e.g., Al, Mg, Ca, etc.), mainly Sources from various forms of soil dust, unorganized emissions, road dust, and human agricultural and forestry activities; pollution elements (such as Pb, As, Cd, etc.), mainly from various industrial processes, etc.^43^. Although small amounts of elements are required for life, most are not considered essential and some of these inorganic elements are neuro disruptors with the ability to cross the blood–brain barrier and these elements are very toxic even at very low concentrations. Studies have shown that circulating heavy metal levels reflect exposure to multiple heavy metals from both natural and anthropogenic sources, that airborne heavy metals will increase contamination of agricultural products and water sources, which will increase intake of toxic elements that will increase circulating heavy metal levels in humans and may lead to genetic mutations or long-term health effects^44^, and that unbalanced circulating metal levels may affect the body's dynamic balance and biological processes and contribute to the development of various diseases, including IS^45^. Among environmental pollutants, toxic heavy metals and metalloids are the most dangerous because they are also not biodegradable and tend to accumulate in the environment, with As, Cd, Pb, and Hg usually being the focus of attention based on their high toxicity^46^.

As previously mentioned, Zhou et al. ^20^.found that metal components such as Cu and As in PM_2.5_ were associated with decreased cardiorespiratory fitness, and this study suggests that attention should be paid to the effects of inorganic elements in PM_2.5_, especially heavy metal elements, on ischemic stroke. Lippmann et al. ^47^.reported that Ni concentrations in road dust (RD) were associated with a dramatic increase in heart rate and heart rate variability in mice, as well as increased mortality in humans. Taken together, these two studies suggest that we should pay attention to the hazards of the relevant heavy metal element components of PM_2.5_ on IS and conduct relevant studies to explain their pathogenic mechanisms. Lin et al.^48^ selected patients with AIS within 1 week of first onset as the experimental group, and included 33 patients with AIS; the healthy control group was participants without a history of stroke or chronic disease except hypertension, and included 39 healthy controls. Serum Pb, Hg, As, and Cd levels were measured in subjects in the experimental and control groups; all participants received an infusion of 1 g of calcium edetate disodium (EDTA), and urine samples were collected 24 h after EDTA infusion to determine the heavy metal levels in the urine. This study aimed to investigate the relationship between heavy metal levels of Pb, Hg, As, and Cd in patients with AIS. It was found that serum and urine Hg levels were lower in patients with AIS, while the differences in other heavy metal levels between the two groups were not statistically significant, which provides new evidence for the dysregulation of heavy metals in patients with AIS. This experiment showed a close correlation between the dysregulation of some heavy metal levels in the human body and the onset of AIS, thus suggesting whether the heavy metals in our PM_2.5_ may trigger the onset and development of IS by influencing the heavy metal levels in the human body, which deserves further study and exploration. Inorganic Pb is known to be considered a probable carcinogen by the International Agency for Research on Cancer, and exposure to Pb in adults can raise blood pressure and potentially increase the incidence of heart disease and stroke. Medina-Estévez et al. ^49^ designed a case–control study to investigate the role of 45 inorganic elements as stroke correlates in 92 patients and 83 controls and found that Pb was positively associated with stroke in both univariate and multivariate analyses. However, this experiment is only a preliminary investigation of the distribution of inorganic elements in stroke patients, and further studies are needed to elucidate potential sources of exposure and to reveal the mechanisms of action of the identified elements in stroke prevalence and prognosis. As previously described, Zanobetti et al. ^24^ evaluated the association between daily PM_2.5_ and hospital admissions for diseases such as CVD in 26 communities in the United States during 2000–2003, intending to explore whether PM_2.5_ chemistry altered the association between cause-specific hospital admissions and PM_2.5_. The study found that for every 10 μg/m^3^ increase in PM_2.5_ concentration, CVD increased by 1.89%; the association between PM_2.5_ and CVD admissions was significantly altered for high-quality Br, Cr and Ni. The study showed that higher masses of Br, Cr, and Ni in PM_2.5_ significantly increased their effect on CVD admissions. As is a naturally occurring non-metallic element and one of the most abundant elements in the earth's crust. Human exposure to As is mainly through drinking contaminated water, followed by respiratory inhalation and dermal contact. As exposure is associated with the development of vascular diseases, including stroke, ischemic heart disease, and peripheral vascular disease, but the toxicokinetics of As are not nearly the same, and individuals are affected differently due to time of exposure, route of ingestion, physicochemical characteristics of the compound and the biological species affected^50^. Evidence suggests that high and moderate-high As intake leads to oxidative stress, inflammation, and vascular endothelial dysfunction, all of which are associated with an increased risk of CVD, particularly stroke, hypertension, myocardial infarction, carotid intima-media thickness, ventricular arrhythmias, and peripheral arterial disease; low-level As concentrations are associated with stroke, elevated systolic, diastolic, and pulse pressures, and coronary heart disease events^51^. Subhani et al. ^52^ study to assess As levels and human exposure risk in outdoor dust from different land use settings (i.e. rural, industrial, and urban) in Punjab, Pakistan, found higher As concentrations in all sample types (i.e. dust, hair, and nails) collected from industrial sites, followed by urban and rural; human risk assessment was also conducted through contaminated dust exposure, which indicated that dust ingestion was the main route of As contamination in the concerned population in all studied land use settings, followed by inhalation and dermal exposure. In conjunction with this study, which reflects that dust exposure is a major source of human As burden and may cause some adverse health effects, the results of this study will help to assess the risk to human health from As pollution due to dust exposure in different regions of the country. As is readily enriched in PM, especially in PM_2.5_, and children and adults may suffer adverse effects through inhalation of As in PM_2.5_^53^. Zhang et al.^54^ used a time-series study approach to investigate the association of particulate matter and 35 components of PM_2.5_ in five size ranges of 0.01 to 2.5 µm with daily emergency room visits for stroke in Shanghai, China, from 2014 to 2019, to assess the association of particulate matter and various components of PM_2.5_ separated by size with daily emergency room visits for stroke. The study found a significant positive correlation between particulate concentration and daily emergency room visits for the size range of 0.01 to 0.3 µm, with the strongest association occurring in the size range of 0.05 to 0.1 µm; At the same time, 11 of the 35 components were identified as having strong correlation, these components are OC, EC, Mg, SNA, Cu, Mn, Pb and Zn. In summary, this study suggests that ultrafine particulate matter and some PM_2.5_ components (i.e., carbonaceous fraction, inorganic ions, and some elements) may be the major contributors to the additional risk of PM_2.5_-induced stroke. Wang et al.^55^ conducted a time-series study to explore the relationship between 27 components of PM_2.5_ and IS hospitalization in Shanghai, China, from 2014 to 2016. The study aimed to identify fine particulate matter (PM_2.5_) air pollution components that have a critical impact on IS to understand the potential biological mechanisms and develop air pollution control policies. It was found that IS was significantly correlated with EC and several elemental constituents (Cr, Fe, Cu, Zn, As, Se, and Pb) at a lag of 1 day. The study indicates that suggests that EC and some metal elements may be the main cause of the risk of IS hospitalization due to short-term PM_2.5_ exposure.

### Organic matter

Organic matter is an important component of PM_2.5_, and its variety is complex, in general, organic pollutants are more hazardous than inorganic pollutants^56^. The organic components in PM_2.5_ are divided into insoluble organic compounds such as aliphatic hydrocarbons, PAHs, polycyclic aromatic ketones, and water-soluble organic compounds such as alcohols, carboxylic acids, phenols, keto acids, and hydroxylamines according to their water solubility. Widely studied organics are PAHs, a group of more than 100 chemical substances consisting of only carbon and hydrogen and composed of two or more aromatic rings^57^. PAHs are formed by the incomplete combustion or pyrolysis of fossil fuels and biomass, as well as by the release of petroleum products and natural sources^58^. PAHs are classified as persistent organic pollutants (POPs), which are persistent, toxic, and ubiquitous compounds that can move long distances and bioaccumulate in the food chain as lipophilic compounds, causing damage to organisms and thus causing disease^59^.

As previously mentioned, Liang et al. ^36^conducted a prospective cohort study of 90,672 adults aged 18 years or older from 161 districts and counties in China from 2010 to 2017, aiming to investigate the association between long-term exposure to PM_2.5_ and its components and CVD mortality in China and to assess the long-term cardiovascular effects of PM_2.5_ and its components, which found that in the general population in China, long-term exposure to An association was found between PM_2.5_ and its components and increased risk of cardiovascular mortality; also PM_2.5_ component hazard studies showed that PM_2.5_ components from fossil fuel combustion (i.e., organic matter, etc.) showed a greater hazard ratio than total PM_2.5_ mass. In their study, Yao et al. ^60^addressed the morphological distribution of volatile organic compounds (VOCs), such as alkanes, olefins, and aromatic and carbonyl compounds, in motorcycle exhaust; some air toxics emitted by motorcycle engines were also studied. This provides a theoretical basis for the origin of PM_2.5_-related components in the air and shows the environmental pollution from the exhaust of vehicles, especially motorcycles. PAHs are potent atmospheric pollutants found in oil, coal and tar deposits and are a byproduct of smoking, indoor and outdoor fuel combustion and food grilling^61^. Motorcycle exhaust also contains gaseous PAHs that are known to be toxic and genotoxic, and, as the Cerna ^62^study shows, gaseous PAHs whose toxicity is strongly concentration-dependent and therefore more significantly toxic than solid PAHs^63^, because gaseous PAHs are more readily absorbed by the body than solid PAHs. In people exposed to PAHs in the workplace or ambient air, studies have found that PAHs are associated with elevated levels of DNA adducts (e.g., PAH-DNA adducts) and p53 mutations^64^. Chang et al.^65^ analyzed the toxicity of PAHs in motorcycle exhaust by dose–response using E. coli DH5α strain and found that PAHs ranked first and were the most toxic compared to other pollutants in motorcycle exhaust, such as VOCs. In summary, PAHs, especially gaseous PAHs, may damage the human body directly through their toxicity and also through interaction with DNA, inducing genetic mutations and producing long-term damage. This may explain the potential cause of AIS damage in IS patients exposed to PAH contamination in PM_2.5_ and the occurrence of ischemic stroke after leaving the pollutant exposure. Preclinical studies have reported a positive association between exposure to PAHs and oxidative stress and atherosclerosis^66^. According to previous studies, there is a positive association between occupational exposure to PAHs and CVD morbidity and mortality^67^. Alshaarawy et al.^68^ in the general US population similarly showed a moderate correlation between urinary PAHs biomarker levels and CVD (IS, etc.), further hypothesizing the pathogenesis of CVD, e.g., IS, coronary artery disease, which is mainly through a common mechanism, i.e., atherosclerosis. Guo et al. ^69^ used PM samples collected in different seasons to treat Wistar rats and measured seasonal changes in rat cortical brain ischemia-related markers, including endothelial function endothelin-1 (ET-1), endothelial nitric oxide synthase (eNOS), inflammatory marker levels inducible nitric oxide synthase (iNOS), cyclooxygenase-2 (COX-2), interleukin-1β (IL-1β), and intercellular adhesion molecule 1 (ICAM-1) and subsequent neurological impairment. The results showed that PM exposure induced endothelial dysfunction, inflammatory response, and neurological impairment similar to IS with seasonal dependence, with the most pronounced damage in winter samples; in addition, the most severe damage to ischemic neurons caused by PM in winter was similarly confirmed by establishing an in vitro ischemic neuron model. Although the samples used by the investigators in this study were PM_10_, the chemical composition of the samples was examined and analyzed for correlation with the aforementioned biomarkers, and it was found that PM in winter had higher PAHs (PAHs) and carbon load, both of which were substances that played a major role in ischemic-like brain injury in samples from different seasons. As previously described, Zhang et al.^21^ obtained city-specific daily numbers of hospitalizations for CVD in 18 cities in China between 2014 and 2017 through the National Basic Medical Insurance Database for Urban Employees and the Beijing Municipal Health and Family Planning Commission Information Center database, and collected directly measured PM_2.5_ components, including ions and PAHs, through the China Environmental Public Health Tracking System. The aim was to investigate the relationship between short-term exposure to PM_2.5_ components and hospitalization for CVD. PAHs were found to be associated with IS, but not with ischemic heart disease. In summary, for the subtypes of CVD, the risk of hospitalization varied depending on exposure to different PM_2.5_ components, with PAHs being associated with IS only. Lim et al.^70^ conducted a case–control study based on the Korean Cancer Prevention Study-II identifying 526 control group members and 111 stroke cases to assess the relationship between serum POP_S_ levels and stroke risk. It was found that elevated serum POPS levels were associated with an increased risk of stroke, particularly IS. Lee et al.^71^ measured 21 POPs (including 16 PCB congeners, 3 organochlorine pesticides, 1 brominated diphenyl ether, and 1 dioxin) in the plasma of 898 participants aged 70 years based on the Uppsala Prospective Vascular Survey of the Elderly, with the aim of assessing whether plasma concentrations of the selected POPs predicted stroke in the elderly. Increased plasma concentrations of PCBs, organochlorine pesticides and organochlorine diphenyl ethers were found to be associated with an increased risk of developing or progressing to stroke in the elderly. Taken together, previous experimental and epidemiological studies suggest that POPs may play a fundamental role in the development of stroke.

### Microorganisms

The vast majority of the harmful components of PM_2.5_ are the chemicals mentioned above. In addition to these, there are a few biologically relevant substances in PM_2.5_, including living organisms (such as bacteria, viruses, fungal spores, pollen, tiny plant or animal fragments, etc.) and dead microorganisms or their related components (such as endotoxins and β-glucans, etc.)^72^. The respirable microorganisms in PM_2.5_ are known to cause the spread of various allergies and respiratory diseases^73^. It has also been shown that acute and chronic microbial infections can lead to large vessel thrombosis and atherosclerosis as well as arterial entrapment, e.g. bacterial meningitis can lead to cerebrovascular inflammation and thrombosis; herpes zoster virus can invade the arterial wall and lead to to to granulomatous and necrotizing vasculitis; Candida albicans, Aspergillus fumigatus or Cryptococcus neoformans can invade cerebral arteries and cause fungal meningitis; helminth infections such as cysticercosis or neurogenic trichinosis can lead to cerebral vasculitis or arterial compression^74^. This suggests that microorganisms, either independently or in association with traditional vascular risk factors, can contribute to brain injury through pathological processes such as atherosclerosis, hematological alterations (leading to a hypercoagulable state), and endothelial dysfunction, which may be a trigger for IS. The most studied about biomass and IS are microorganisms such as bacteria, viruses and fungi, and the rest of biomass, such as pollen, is less studied. Ample evidence from studies with different methodological designs suggests that acute infection with one or more bacteria and/or viruses is an important independent trigger for stroke^75^, where viral infections can affect the peripheral and central nervous system and lead to an increased risk of stroke^76^. Fungal spore levels are usually higher in rural areas but may also be abundant in urban areas^77^. Variations in fungal concentrations depend on changes in PM concentrations, meteorological conditions, and solar radiation, and there is also an association between changes in their concentrations and stroke onset^78^. Therefore, treatment strategies aimed at preventing infections with pathogenic microorganisms may also prevent stroke and deserve further investigation.

PM pollution poses a significant threat to public health, and while the physical and chemical properties of PM pollutants have been extensively studied, much less is known about respirable microorganisms, among the various hazards of PM pollutants, microorganisms in PM_2.5_ are thought to be responsible for the spread of various allergies and respiratory-related diseases. Using a metagenomic approach to analyze the microbial composition of PM pollutants during the severe haze event in Beijing in January, Cao et al.^73^ found that airborne microorganisms, including bacteria, archaea, fungi, and dsDNA viruses, could be identified at the species level at sufficient sequencing depth. The study showed that most respirable microorganisms are soil-associated, and their relative abundance increases with increasing PM pollution concentrations. Lee et al.^79^ conducted a search of databases for literature on IS up to October 22, 2020, and systematically reviewed the relationship between human microbiota and IS, showing that different microbial components may contribute to IS and suggesting that modulation of flora ratios may be a potential target for IS therapy. In summary, we should start to think and study the effect of microorganisms in PM on IS and explore the connection between the two to provide prevention and treatment. Frequent throughout the year in some parts of the world, dust storms originate in many desert areas or arid regions of the world and have an impact not only on the health of humans living there, but also on people in downwind environments. Dust becomes finer with distance from the source; for example, in the case of the Sahara Desert, the coarsest dust (over 70 μm) occurs close to the Sahara Desert itself, while dust farther downwind tends to be 5 to 30 μm or less in diameter^80^. Sand and dust are dominated by SiO_2_ and Al_2_O_3_, other important components are Fe_2_O_3_, CaO, and MgO, It also contains a large amount of salts, organic components, etc. Crucially, it can also transport pathogens and anthropogenic pollutants, All these potential sand and dust components may negatively affect human health in the local area and downwind environment, thus posing a health risk to the population in the environment in which it is located^81^. The greatest risk factors regarding dust storm exposure are the frequency of exposure, the concentration and composition of particulate matter, and especially the microbial composition of dust, such as bacterial lipopolysaccharides, fungi, and viruses, to directly affect human health. In his article, Goudie^82^ discusses PM_10_ and PM_2.5_ load data from desert dust events, as well as various pollutants (heavy metals, pesticides, etc.) and biological components (spores, fungi, bacteria, etc.), finding that at lower particle concentrations, finer particles (PM_2.5_) make up a larger percentage of total particles; that sand and dust carry and transport biological material, including bacteria, pollen spores, fungi, and viruses, which can survive long-distance transport across the globe, all of which have potential implications for the incidence of associated diseases; particle size is a major determinant of where particles remain in the respiratory tract when inhaled, with particles < 12.5 μm tending to penetrate the gas exchange zone of the lungs and ultrafine particles (< 100 nm) potentially crossing the lungs to affect other organs and potentially lead to cardiovascular consequences, and the human health effects of dust include respiratory diseases (including asthma, bronchitis, pneumonia, allergic rhinitis and silicosis), CVD (including stroke), etc. The best-known human pathogen associated with desert dust storms is Fungus Coccidioides immitis^83^. In addition, the closest known link to microbial-induced neurological disease in dust storms is the outbreak of meningitis (mainly caused by Neisseria meningitides infection) that occurred in the "meningitis belt" of North Africa, where dust storms are thought to cause nasopharyngeal mucosal abrasion and promote infection through inhalation of dust particles, bringing Neisseria meningitides cells residing in the mucosa into contact with the underlying tissues and blood, thus causing infection; a complementary hypothesis suggests that Neisseria meningitidis adheres to inhaled dust particles, enters the lungs, and directly attacks lung tissue causing damage that indirectly leads to neurological damage and meningitis^84^. Although we know that dust contains pathogens, the risks associated with it remain largely unknown in most cases and still need to be explored further. Bacterial lipopolysaccharides can also directly affect human health through inhalation. Lipopolysaccharides are cell wall components of Gram-negative bacteria and are commonly found in dust, and animal inhalation tests have shown that exposure to the lipopolysaccharide component of PM_2.5_ is associated with the development of IS^85^. Neocoronary pneumonia caused by the SARS-CoV-2 virus has triggered a global health crisis, and although the initial major concern focused on the risk of acute respiratory distress syndrome with high mortality, cardiovascular symptoms are increasingly reported as the number of cases gradually increases^86^. Katsoularis et al.^87^ quantified the risk of IS associated with novel coronavirus pneumonia by analyzing all cases of novel coronavirus pneumonia based on all patients with novel coronavirus pneumonia in Sweden between February 1 and September 14, 2020, using an own-control case series and matched cohort study approach. This literature suggests that the presence of viruses associated with airborne particulate matter, such as SARS-CoV-2 virus, may influence the development of IS, and this should be taken into account and studied.

## Summary and prospect

**Figure 1:**
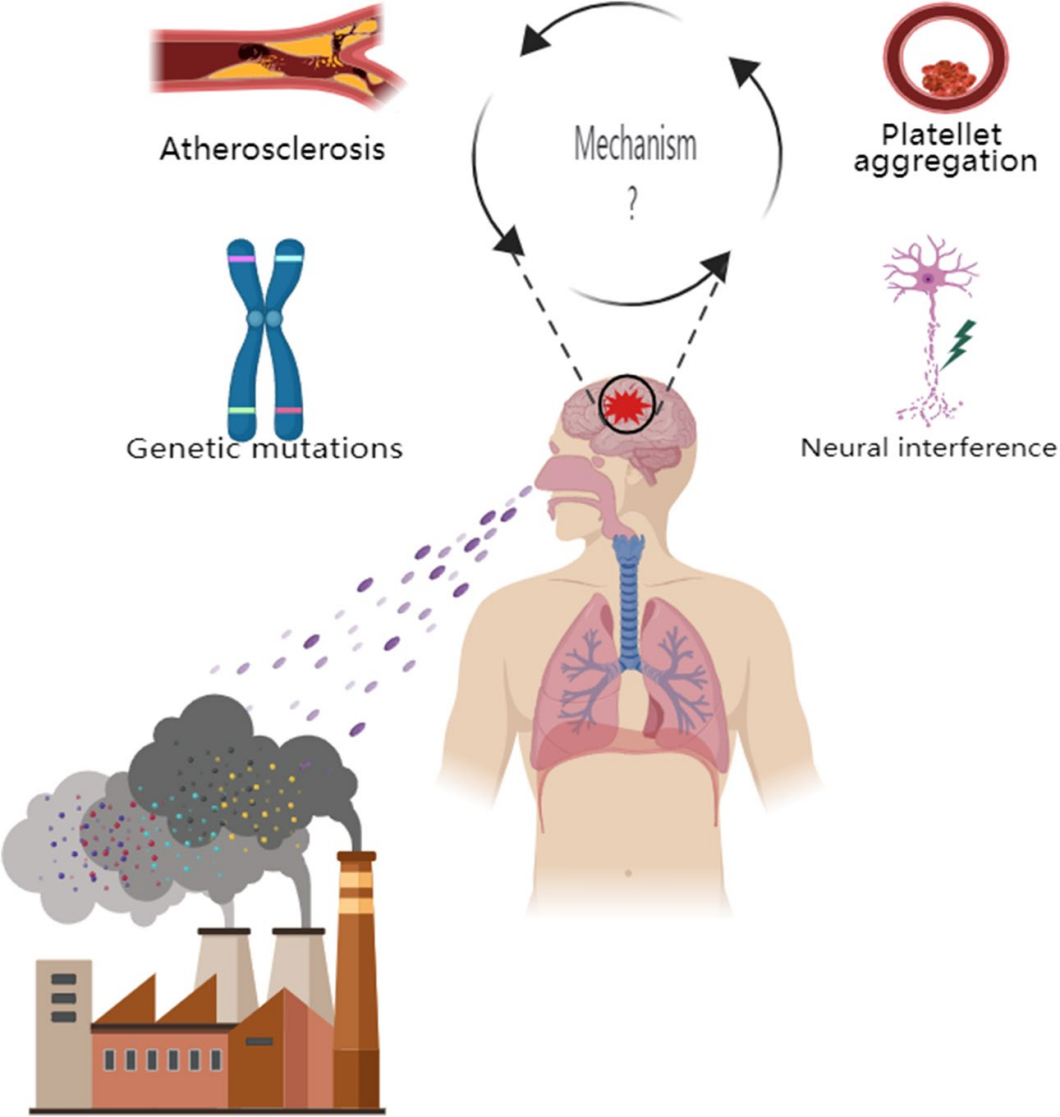
The main pathways and possible mechanisms by which PM_2.5_ and its different components affect ischemic stroke. The different colored balls represent different components of PM_2.5_, which affects ischemic stroke mainly through the respiratory pathway. The possible mechanisms of ischemic stroke involve the pathways of atherosclerosis, platelet aggregation, genetic mutation, and neural interference et al.

PM_2.5_ exposure is one of the risk factors for IS disease, and both short-term and long-term PM_2.5_ exposure can increase the risk of IS morbidity and mortality. Assessing the role of different components of PM_2.5_ in the development of IS has important environmental implications not only for the source management of PM_2.5_ but also, more importantly, for the prevention and treatment of IS, which has important clinical implications. For water-soluble inorganic ions, which account for a relatively large proportion of the total mass of PM_2.5_, the three salts formed by SO_4_^2−^, NO^3−^ and NH_4_^+^ with the ions in question, i.e., SNA, have received the most research attention, in addition to some of the studies that have focused on Na^+^ and NO_X_; the possible mechanisms by which water-soluble inorganic ions affect ischemic stroke involve chronic inflammation, oxidative stress, platelet aggregation and vascular endothelial cell injury among other pathways^88^. For the carbon-containing components, more research attention has been given to OC and EC (also known as BC), but more research has been done on EC, possibly because the OC component overlaps with the organic component discussed subsequently, and researchers may prefer to categorize them as organic; possible mechanisms by which carbon-containing components affect ischemic stroke involve pathways such as accelerating plaque formation and thus atherosclerosis, affecting cardiac autonomic function, and contributing to platelet aggregation. cardiac autonomic function, and platelet aggregation, among other pathways^89^. For inorganic elements, There is a wide variety of them, and more attention has been paid to the heavy metal elements among them; The current study mainly analyzes the content and percentage of various types of inorganic elements in the blood of IS patients, although it is not a study for the direct exposure to PM_2.5_, it is partly persuasive as the circulating levels of heavy metals reflect the exposure to a wide range of heavy metals of both natural and anthropogenic origins; the influence of inorganic elements on the Possible mechanisms of ischemic stroke involve pathways such as neural disturbances, genetic mutations, and perturbation of the body's dynamic homeostasis and biological processes^90^. For organics, which are more harmful to the pathogenesis of IS than the other components of PM_2.5_, the organics that have been widely studied are PAHs, which are classified as POPs, in addition to other organic pollutants such as alkanes, olefins, and aromatics, which have been the subject of some studies; the possible mechanisms by which organics affect ischemic stroke involve pathways that involve oxidative stress, lead to atherosclerosis, and induce genetic mutations^91^. For pathogenic microorganisms, The most researched pathogenic microorganisms are bacteria, viruses, and fungi, In addition to this, some studies have also focused on the harmful effects of microbe-related components in IS disease, such as lipopolysaccharides, etc., and more and more studies have begun to focus on the effects of SARS-CoV-2 virus on IS in recent years due to the impact of the epidemic of SARS-CoV-2 virus; Possible Mechanisms by Which Pathogenic Microorganisms Affect Ischemic Stroke Pathways involving inflammatory damage, causing vascular endothelial dysfunction and keeping blood in a hypercoagulable state^92^ (Table 1). There are three general problems with the current studies on the different components of PM_2.5_ and IS: first, the composition of PM_2.5_ is not sufficiently discussed, focusing only on specific components of PM_2.5_, resulting in an incomplete study of PM_2.5_-related components and IS; second, there are fewer studies on the interaction between PM_2.5_ components, focusing only on the effect of a single component on IS and not on whether PM_2.5_ components reinforce or mitigate each other's effects on IS; third, there are fewer studies on the mechanisms related to the development of IS by different components of PM_2.5_, focusing only on the association between exposure to different PM_2.5_ components and IS outcomes, and there is a lack of research on its physiopathological mechanisms, such as oxidative stress, inflammation, vascular dysfunction and remodeling, activation of prothrombotic pathways, autonomic dysfunction, and blood pressure changes^93^ (Fig. 1).

**Table 1:** Specific components of PM_2.5_ and possible mechanisms for the association with ischemic stroke.

	Different components of PM_2.5_									
	Water-soluble inorganic ions (water-soluble anions and cations)			Carbon-containing fractions		Inorganic elements		Organic matter		Microorganisms
Specific components	Sulfate–nitrate–ammonium salts (SNA)			Organic carbon (OC)		Heavy metal elements		Insoluble organic compounds		Living organisms
	Cations			Primary organic carbon (POC)		Major elements (Al/Mg/Ca etc.)		Polycyclic aromatic hydrocarbons (PAHs)		Bacteria
	Ammonium (NH_4_^+^)			Secondary organic carbon (SOC)		Minor elements (Ti/Cu/Zn etc.)		Aliphatic hydrocarbons		Viruses
	Calcium (Ca^2+^)			Elemental carbon (EC) (light-absorbing carbon or black carbon)		Trace elements (Be/Ag/Cd etc.)		etc.		Fungal spores
	etc.			Carbonate carbon (CC)		Other inorganic elements		Water-soluble organic compounds		etc.
	Anions					Bromine (Br)		Carboxylic acids		Dead microorganisms or their related components
	Sulfate (SO_4_^2−^)					etc.		Phenols		Endotoxins
	Nitrate (NO_3_−)							etc.		β-glucans
	etc.									etc.
Possible mechanisms	Oxidative stress Chronic inflammation Platelet aggregation Vascular endothelial cell injury etc.			Atherosclerosis Platelet aggregation Accelerated plaque formation Cardiac autonomic dysfunction etc.		Genetic mutations Neural interference Disturbance of the body's dynamic equilibrium and biological processes etc.		Bioaccumulation Atherosclerosis Oxidative stress induced mutations etc.		Atherosclerosis Inflammatory injury Vascular endothelial dysfunction Hematologic changes (hypercoagulability) etc.

In general, on the one hand, there is an association between the different components of PM_2.5_ and IS. Therefore, studying the composition of PM_2.5_, identifying the components of PM_2.5_ that are most hazardous to human health, and then assessing the exposure of different components of PM_2.5_ in the population will help to provide a reasonable explanation for the occurrence and development of IS. On the other hand, compared to well-documented IS risk factors such as smoking, poor diet, and lack of physical activity, PM_2.5_ exposure is a potentially modifiable risk factor independent of changes in individual behavior, and even the current authoritative WHO guidelines do not guarantee optimal health protection, even below the maximum PM_2.5_ exposure concentration currently recommended by WHO. Therefore, for individuals, it is recommended that people should be aware of air quality and reduce their exposure to PM_2.5_; for the research community, because PM_2.5_ research is increasingly interdisciplinary, understanding its sources, composition, effects, and solutions requires integrated capabilities, and therefore requires extensive knowledge and information on chemistry, physics, geographic information, earth systems, meteorology, and biology to discover which PM_2.5_ component exposures are the most harmful, to deal with the complex PM_2.5_ component exposures on population health effects, and ultimately find control strategies; for government organizations, recognizing that improving air quality has considerable public health implications for reducing the burden of IS, and thus considering certain measures to enact more targeted air quality standards or guidelines, including PM chemical composition, other characteristics, or specific sources of PM, to control air pollutant emissions and protect public health and life safety.

## Data Availability

The datasets used and/or analyzed during the current study are available from the corresponding author on reasonable request;
